# Age-dependent genetic regulation of osteoarthritis: independent effects of immune system genes

**DOI:** 10.1186/s13075-023-03216-2

**Published:** 2023-12-01

**Authors:** Jacob Kenny, Benjamin H. Mullin, William Tomlinson, Brett Robertson, Jinbo Yuan, Weiwei Chen, Jinmin Zhao, Nathan J. Pavlos, John P. Walsh, Scott G. Wilson, Jennifer Tickner, Grant Morahan, Jiake Xu

**Affiliations:** 1 School of Biomedical Sciences, University of Western Australia, Crawley, WA 6009 Australia; 2 Department of Endocrinology & Diabetes, Sir Charles Gairdner Hospital, Nedlands, WA Australia; 3 Australian Institute of Robotic Orthopaedics, Crawley, WA Australia; 4 Research Centre for Regenerative Medicine, and Guangxi Key Laboratory of Regenerative Medicine, Guangxi Medical University, Guangxi, China; 5 Medical School, University of Western Australia, Crawley, WA Australia; 6 Department of Twin Research & Genetic Epidemiology, King’s College London, London, UK; 7 Centre for Diabetes Research, Harry Perkins Institute for Medical Research, Nedlands, WA Australia; 8 Shenzhen Institute of Advanced Technology, Chinese Academy of Sciences, Shenzhen, 518055 China

**Keywords:** Osteoarthritis, Genome-wide association, Genetics, Immune regulation

## Abstract

**Objectives:**

Osteoarthritis (OA) is a joint disease with a heritable component. Genetic loci identified via genome-wide association studies (GWAS) account for an estimated 26.3% of the disease trait variance in humans. Currently, there is no method for predicting the onset or progression of OA. We describe the first use of the Collaborative Cross (CC), a powerful genetic resource, to investigate knee OA in mice, with follow-up targeted multi-omics analysis of homologous regions of the human genome.

**Methods:**

We histologically screened 275 mice for knee OA and conducted quantitative trait locus (QTL) mapping in the complete cohort (> 8 months) and the younger onset sub-cohort (8–12 months). Multi-omic analysis of human genetic datasets was conducted to investigate significant loci.

**Results:**

We observed a range of OA phenotypes. QTL mapping identified a genome-wide significant locus on mouse chromosome 19 containing *Glis3*, the human equivalent of which has been identified as associated with OA in recent GWAS. Mapping the younger onset sub-cohort identified a genome-wide significant locus on chromosome 17. Multi-omic analysis of the homologous region of the human genome (6p21.32) indicated the presence of pleiotropic effects on the expression of the *HLA − DPB2* gene and knee OA development risk, potentially mediated through the effects on DNA methylation.

**Conclusions:**

The significant associations at the 6p21.32 locus in human datasets highlight the value of the CC model of spontaneous OA that we have developed and lend support for an immune role in the disease. Our results in mice also add to the accumulating evidence of a role for *Glis3* in OA.

**Supplementary Information:**

The online version contains supplementary material available at 10.1186/s13075-023-03216-2.

## Key messages

What is already known about this subject?

• Genetic factors play a role in OA development, with loci identified via genome-wide association studies (GWAS) estimated to account for 26.3% of the disease trait variance in humans.

What does this study add?

• We identified two genetic loci in mice associated with the development of spontaneous knee OA.

• Regulation of *HLA-DPB2* expression by DNAm sites may have a role in the development of knee OA, providing supporting evidence for a potential immune role in the disease.

How might this impact clinical practice?

• Further investigation into variations in *GLIS3* and *HLA-DPB2* and their functions may provide new diagnostic tools and potential treatment targets for spontaneous knee OA.

**Supplementary Information:**

The online version contains supplementary material available at 10.1186/s13075-023-03216-2.

## Introduction

Osteoarthritis (OA) is the most common joint pathology. It affects mainly load-bearing joints such as the hip and knee and is a leading cause of pain and disability [1, 2]. There are more than 250 million sufferers worldwide of knee OA [3]. While OA affects all age groups, cases become more prevalent after 50 years of age in men and 40 years in women [4, 5]. It is known that OA is associated with ageing; however, it is not considered to be a natural component of the ageing process [6].

The current diagnostic criteria for knee OA are by clinical assessment, evaluation of radiographic imaging and subjective self-assessment, which may not correlate with clinical or radiographic findings [2, 7–9]. The limitations of these techniques, in particular the inability to identify patients in the early stages of knee OA, suggest that new and more specific screening methods are required to identify patients in the early stages of knee OA.

There is evidence that there is a heritable component to the development of OA; the sibling recurrence-risk ratio has been estimated to be 2.08–2.31 for radiographic knee OA and 4.27–5.07 for radiographic hip OA [10]. A study of 130 identical and 120 non-identical twins suggested that 35 to 65% of hand and knee OA was due to genetic factors [11], while genetic contributions to hip OA in women were estimated at 60% [12]. Studies performed with inbred mouse strains such as STR/ort have observed the development of spontaneous OA phenotypes [13], further indicating a strong genetic basis for the disease. Another study has shown that screening for a collection of 23 single nucleotide polymorphisms (SNPs) associated with OA phenotypes in patient cohorts can be used as an accurate prognostic tool [14].

Investigation into the genetics of OA has traditionally followed the common disease-common variant hypothesis [15]. Genome-wide association studies (GWAS) investigating OA, such as those conducted by the UK Biobank [16, 17] and the arcOGEN Consortium [18, 19], have been highly successful in identifying many new genetic loci associated with the disease; more than 60 loci have now been identified as associated with OA, and collectively, these account for approximately 26% of the trait variance [16]. Despite this success, these studies have some acknowledged limitations, including poorly defined phenotypes and the inability to account for external factors contributing to the disease such as environment, body mass index, diet and lifestyle [16].

Quantitative trait locus (QTL) mapping using mice can overcome several limitations of human GWAS by controlling external factors and can be used to investigate spontaneous OA. The STR/ort mouse model of genetically influenced spontaneous OA develops OA between the ages of 9 and 12 months [13, 20, 21]. A QTL mapping study for OA in the STR/ort mice using a small panel of 122 microsatellite markers successfully identified loci containing known OA-associated genes [22]. Recombinant inbred (RI) mouse strains provided better power and mapping resolution, and they also use SNPs rather than microsatellite markers [23, 24]. The Collaborative Cross (CC) is a large panel of RI mice derived from eight genetically diverse founder strains (A/J, C57BL/6 J, 129S1/SvImJ, NOD/LtJ, NZO/H1LtJ, CAST/EiJ, PWK/PhJ and WSB/EiJ) that collectively encompass close to 90% of common genetic variation found in the mouse genome. Locus mapping of CC strains is conducted by utilising over 150,000 SNP markers distributed throughout the mouse genome to provide high-resolution genome mapping capabilities when compared with previous RI panels [25, 26] and is capable of mapping genes and identifying the actual base changes responsible for traits of interest [27, 28].

Conducting QTL mapping for OA with the CC mice yields many advantages compared to human GWAS. These include the ability to assess individuals of the same genotype at desirable time points, carefully control for many of the environmental factors contributing to the disease and the ability to collect fresh tissue. Reproducibility is high because multiple individuals of the same strain (i.e. same genotype) can be tested, in contrast to humans where (apart from identical twin studies) each person has a different genotype. Furthermore, histological evaluation of the intact murine joint is possible, thus providing a platform to carefully map the genetic component of spontaneous OA in the mouse genome. The results from QTL mapping in mice also enable a more targeted analysis of human genetic datasets, reducing false negatives arising from stringent genome-wide statistical significance thresholds. Such an approach has been used previously to identify genes associated with HDL cholesterol levels [29].

We aimed to discover novel genes and regulatory elements relevant to OA. For this, we applied the OARSI grading method for OA in the CC mice from an adult cohort, spanning 8 to 24 months of age with a subset spanning 8 to 12 months classified as younger onset. We conducted QTL mapping using the OA scores derived from these cohorts to identify loci associated with OA severity. Significant loci were then investigated using multi-omic human genetic data to help characterise the clinical relevance of the findings and identify likely effector genes and biological mechanisms involved in the disease.

## Methods

### Mouse model

The mice screened for OA were derived from the Geniad Collaborative Cross Gene Mine colony, which is bred and housed at the Animal Resources Centre (Murdoch, Western Australia). The CC mice were produced from eight founder strains (A/J, C57BL/6 J, 129S1/SvImJ, NOD/ShiLtJ, NZO/H1LtJ, CAST/EiJ, PWK/PhJ and WSB/EiJ) as previously described [25, 30]. A more detailed explanation of the CC model is explained in Additional file 2: Supplementary Methods. The use of all animals in this study was conducted in accordance with the Australian Code for the Care and Use of Animals for Scientific Purposes, under the approval of the Animal Ethics Committees of the University of Western Australia, and the Animal Resources Centre. The hind limbs were dissected from a total of 275 combined male and female Geniad mice from 50 strains for phenotype screening. After the exclusion of samples with poor tissue conditions and strains comprising only a single sample, the total numbers used for QTL mapping were 222 mice from 43 strains for the adult cohort. The subset of this cohort capturing those with younger onset of OA included 94 mice from 24 strains.

### Histological analysis

The intact left limbs were processed for histological examination of the knee joints. The limbs were fixed in 10% neutral buffered formalin for 24 h, rinsed in PBS and transferred to 14% EDTA for 3–5 days at 37 °C for decalcification, then processed into paraffin wax for sectioning. Each limb was sectioned at 5 μm from a frontal orientation to view the medial and lateral compartments and trimmed to a depth to display the initial tibial attachment of the anterior cruciate ligament (ACL). The sections were collected every 40 μm for observation until the posterior cruciate ligament (PCL) was the dominant feature in the centre of the joint, and the lateral and medial meniscus had lengthened to identify the posterior horn for both menisci. The sections were stained using Safranin O for microscopic analysis.

### Histological scoring

**Fig. 1:**
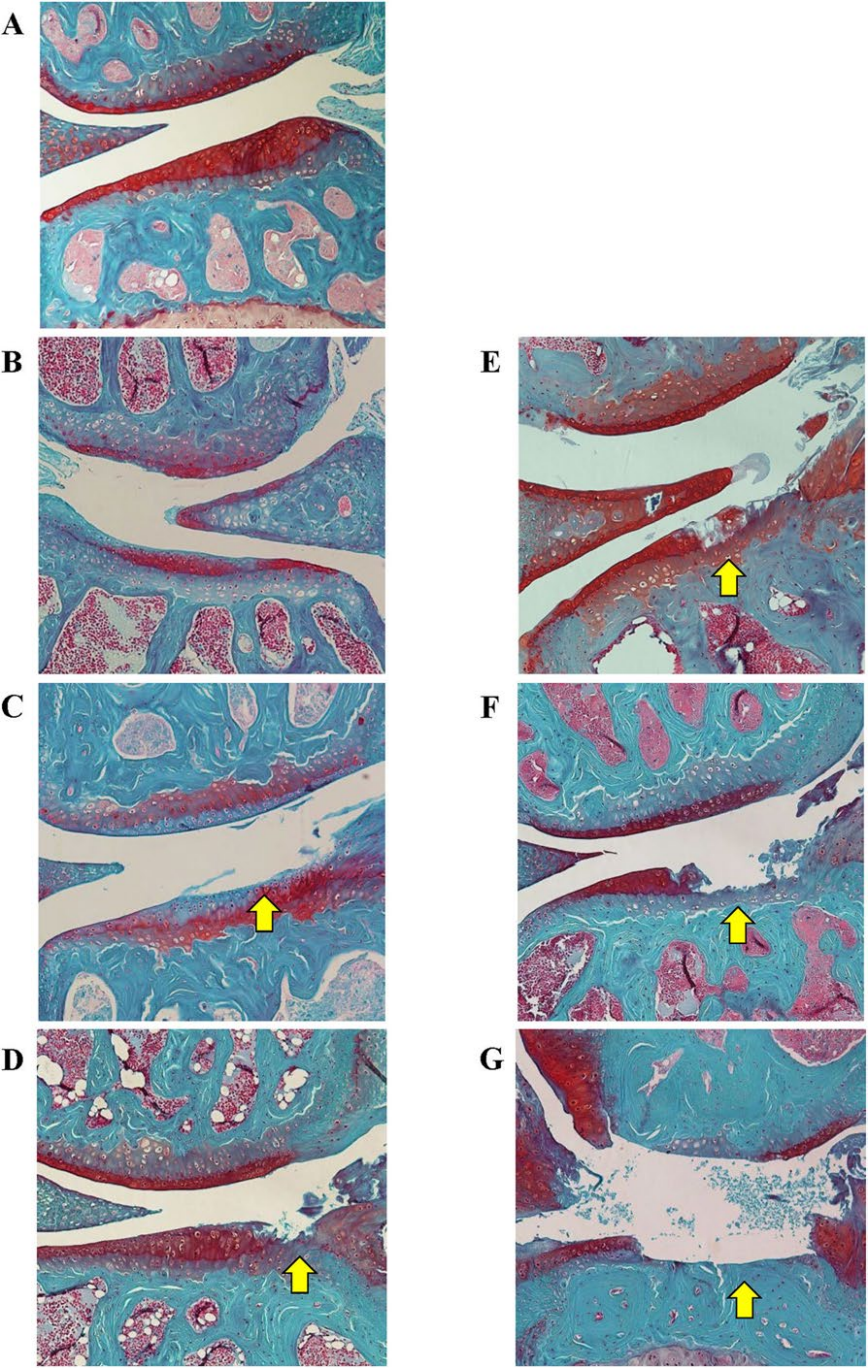
Histological analysis of OARSI scores observed and represented in the CC mice. Medial/lateral knee compartments of CC mice stained with Safranin O and Fast Green. Grade 0 (**A**), grade 1 (**B**), grade 2 (**C**), grade 3 (**D**), grade 4 (**E**), grade 5 (**F**) and grade 6 (**G**). Area corresponding to OARSI score is represented by yellow arrows

Scoring of OA severity was conducted using the Osteoarthritis Research Society International (OARSI) method [31] (Additional file 1: Table S1). Each joint was scored based on the combined scores of the medial tibial plateau (MTP), lateral tibial plateau (LTP), medial femoral condyle (MFC) and lateral femoral condyle (LFC). A subset of samples representing a range of mild to severe OA scores was co-scored by an experienced pathologist for scoring validation (Fig. 1). The scored cohort was assessed as a middle-aged cohort to characterise cases of younger onset OA (ages 8–12 months) and a complete adult cohort (> 8 months).

### QTL mapping

After obtaining the phenotype data, we were able to map relevant genes by correlating phenotypes observed in the CC mice with their genotypes using the GeneMiner software [32] (https://www.sysgen.org/Geniad2/). GeneMiner calculates the contribution of each founder to each observed trait by coefficients (log of odds ratio) of the fit from the logistic/multinominal regression model and using the plotting tools in the DOQTL R package [33]. The dominant founder contributions calculated within the system to each QTL are represented by *P*-values. The SNPs unique to the relevant founder/s at the QTL in question are considered as candidate contributor/s to the phenotype.

Numerical values were assigned to the CC strains based on the median OARSI score observed in their respective phenotype classifications. The data was entered into GeneMiner (version: 19/05/2016) to compare haplotypes of the strains and identify loci that were represented by founder contributions unique to the high-scoring CC strains. The QTL analysis was carried out using both raw data format (i.e. “as is”) and normalised data. Each run generated a QTL plot for each respective parameter or phenotype. GeneMiner output is displayed as a plot with the genomic region on the *x* axis and logarithm of the odds (LOD) score on the *y* axis. Two significance thresholds are represented: the 95th percentile of confidence which is equivalent to *P* < 0.05 (red line) and the 37th percentile of confidence which is equivalent to *P* < 0.63 (yellow line). Peaks that cross the 95th percentile are genome-wide significant, while peaks crossing only the 37th percentile are genome-wide suggestive. This approach to QTL analysis has been successfully applied by others [28, 34, 35].

### Multi-omic analysis of human datasets

Regions of the human genome homologous to significant QTLs identified in the CC mice were subjected to an integrative analysis of human knee OA GWAS results with human whole-blood expression quantitative trait locus (eQTL) and methylation quantitative trait locus (mQTL) data. eQTL studies map associations between genetic variants and gene expression, while mQTL studies identify genetic variants associated with nearby DNA methylation sites. This multi-omic analysis was performed using the Summary-data-based Mendelian Randomisation (SMR) software [36]. This package applies the principles of Mendelian randomisation to summary-level genetic association results to identify DNA methylation (DNAm) sites associated with gene expression and a trait of interest through pleiotropic effects. The software performs an SMR test, which identifies shared association signals in two datasets, followed by a heterogeneity in dependent instruments (HEIDI) test, which compares nearby co-inherited markers to determine whether a single causal variant is underlying the associations (pleiotropy). A significant HEIDI result suggests that heterogeneity exists in the two datasets; therefore, the presence of a single causal variant is considered less likely. We integrated the knee OA GWAS results dataset published by Tachimazidou et al. (*N* = 24,955 cases and 378,169 controls) [17] with the lite versions (including variants associated at *P* < 1.0 × 10^−5^) of the whole-blood expression quantitative trait locus (eQTL) summary data from GTEx V7 (*N* = 393) [37] and peripheral blood methylation quantitative trait locus (mQTL) summary data (*N* = 1980) [38]. For linkage disequilibrium (LD) estimation, we used whole-genome sequence data from 1854 Caucasian individuals [39, 40], and only genes/DNAm sites with a minimum of 1 eQTL/mQTL association at *P* < 5 × 10^−8^ were included in the analyses. For the SMR test (*P*_SMR_), correction for multiple testing was performed using the Bonferroni method, while a significance threshold of *P*_HEIDI_ > 0.01 was used to identify pleiotropic associations.

### Patient and public involvement

Patients and public involvement were not applicable to this study.

## Results

### Total joint OA analysis in adult mice

**Fig. 2:**
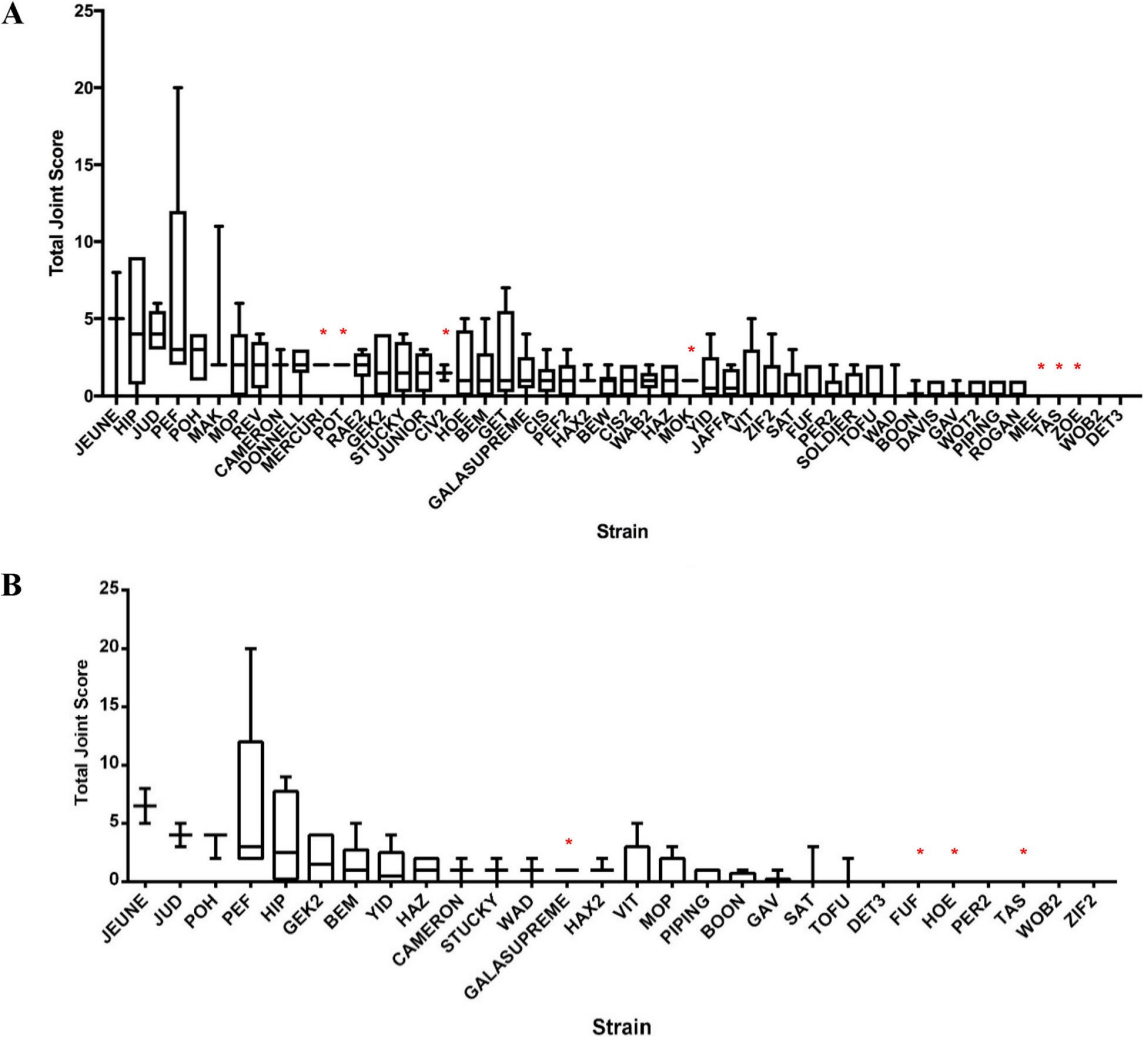
Distribution of OARSI scores of mice per strain (mean, 25/75%, maximum/minimum score). Total joint score of the > 8-month cohort. *n* = 237 (*n* = 222 used for mapping) (**A**). Total joint score of the 8–12-month younger onset cohort. *n* = 101 (*n* = 94 used for mapping) (**B**). Red asterisk represents the strains that were not included in the downstream QTL mapping

We screened 275 male and female mice derived from 50 CC strains, at a minimum age of 8 months. Of these, 237 met the criteria of adequate tissue quality for OARSI scoring and analysis. The joints showed OA severity scores that differed both between (Figs. 1 and 2) and within strains (Fig. 2). Of the strains that satisfied the inclusion criteria, 11.6% showed OA in all individuals. Individuals analysed from the strains JUD, PEF, POH, HAX2 and MAK displayed an appreciable degree of OA. PEF displayed the highest total joint OARSI score with a mean value in the top 10% of scores (Fig. 2). Strains JEUNE and HIP presented with the highest mean values while WOB2 and DET3 displayed no evidence of OA (i.e. an OARSI score of 0) in any of the individuals examined.

### QTL mapping in the CC cohort

**Fig. 3:**
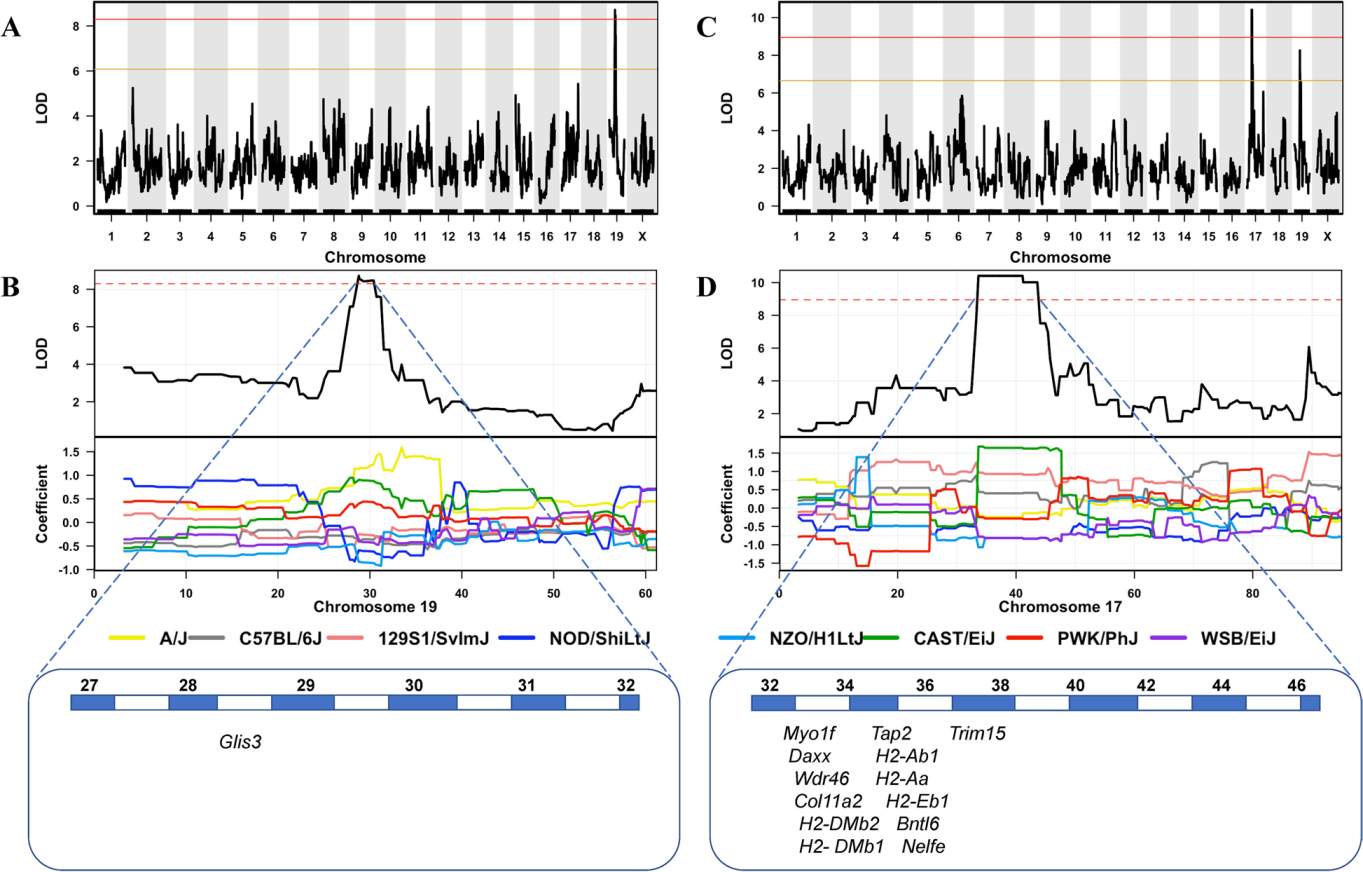
QTL mapping of median OA phenotype scores. Total joint OA measured in the entire cohort (**A**), an expanded view of chromosome 19, highlighting the genome-wide significant peak and associated genes (**B**). Total joint OA measured in the 8–12-month younger onset cohort (**C**), an expanded view of chromosome 17, highlighting the genome-wide significant peak and associated genes (**D**). Red lines indicate a threshold for genome-wide significance, and yellow lines indicate a genome-wide suggestive threshold. Below is a zoomed-in view of the chromosome containing the peak, the founder coefficient demonstrates the founder contributions to the locus and the genes implicated at the locus

To determine the loci associated with OA phenotypes in the CC strains, we conducted QTL analysis of total joint OA in mice ranging from 8 to 24 months old. This used data from 222 individuals of 43 CC strains that met the inclusion criteria of *n* ≥ 3 per strain. In the analysis of median total joint OA score per strain, we observed a QTL at the genome-wide significance threshold located between 27.82 and 31.51 Mbp on chromosome 19 (Fig. 3). Analysis of founder effects showed that the CAST and A/J strains were the main contributors to the phenotype. Of the 23 genes within this interval, the Sanger Mouse Genomes Project database showed that only *Glis3* had polymorphisms specific to the AJ and CAST strains (Additional file 1: Table S3).

### Younger onset OA QTL mapping

To investigate genes that may mediate OA with younger onset (i.e. prior to 12 months), we analysed CC mice aged between 8 and 12 months (Fig. 2). This group contained 24 strains consisting of 94 individuals after exclusion of poor-quality samples and strains with ≥ 2 replicates. QTL analysis of median values of the highest single OA defect in the 8–12-month cohort revealed two prominent loci, one on chromosome 17 between 32.5 and 44.2 Mbp that reached genome-wide significance and another on chromosome 19 between 27.8 and 28.3 Mbp that demonstrated suggestive association (Fig. 3). The chromosome 17 locus included the major histocompatibility complex (MHC), the most gene-rich and polymorphic region of the genome. Again, the CAST founder strain made the greatest contribution to the variation in this trait but both B6 and 129 Sv founder strains also had susceptibility alleles. The Sanger SNP database was searched for SNPs that were shared by these three strains but were different in the other five founders. There were no protein-changing SNPs or indels that had this pattern. Therefore, we also searched the ECCO database [41], comprising ~ 5 million sequences aligned to ~ 300,000 functional elements (such as promoters, enhancers and CTCF binding sites) experimentally identified by the mouse ENCODE project in 19 different tissues [42]. This allows interrogation of sequence variation of functional elements in the CC founder strains. Additional file 1: Table S4 shows a total of 50 SNPs that affect experimentally verified regulatory elements and whose alleles show the correct strain distribution pattern (i.e. shared by B6, 129 and CAST, but different in all other founders). As shown in the table, most of these SNPs affect methylation sites.

### Identification of OA risk and resistance alleles

Since the *Glis3* locus was also suggestively associated with younger onset OA, we considered the interaction between the two loci. Six strains had susceptibility alleles at both loci—five of these had the highest OA scores with an average of 4.1 (ranging from 2.5 to 6.5). The sixth had a score of 1. A total of 18 strains were identified as carriers of an OA resistance allele at *Glis3*. None of the 18 strains with a resistance allele at *Glis3* had severe OA (Additional file 1: Table S2).

Four strains had a susceptible H2 haplotype but a protective *Glis3 allele*; these had a score of 1. Twelve of 14 strains with no susceptibility alleles had no OA at all; two others had a score of 1. We used the non-parametric Fisher’s exact test to test the probability of that result versus no interaction under the null hypothesis. The *P* value obtained (0.0001) indicated that the null hypotheses could be rejected (Table 1).

**Table 1:** Interaction between Chr 17 (*H2*) and Chr 19 (*Glis3*) loci

*Glis3*	*H2*	Severe young OA	Mild/no OA	*P*
R	R/S	0	19	
S	S	5	1	0.00011

*P* value derived from Fisher’s exact test

*R* resistance allele, *S* susceptibility allele

**Fig. 4:**
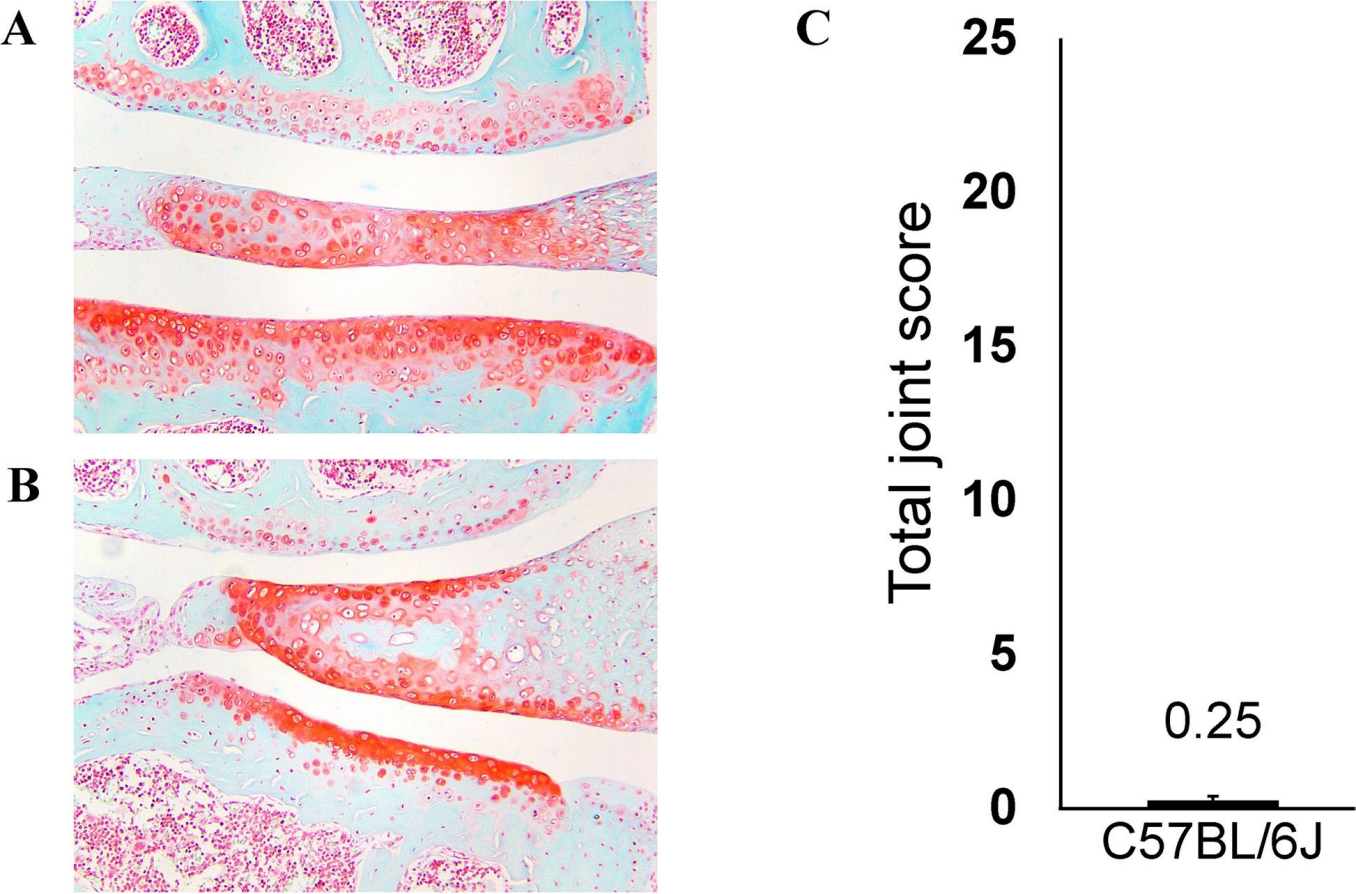
Histological analysis of OA in C57BL/6 J mice aged 8 months. Medial/lateral knee compartments stained with Safranin O/Fast green. Eight-month female (**A**). Eight-month male (**B**). Total joint OARSI score of C57BL/6 J cohort, *n* = 6 (**C**)

The genetic mapping results suggest that the powerful immune contribution of the MHC accelerates disease onset mediated by *Glis3*. In the absence of this MHC affect, the susceptibility alleles of *Glis3* contribute to OA, but this is not evident until an older age of onset. This also suggests that protective *Glis3* alleles can protect against younger onset OA. We tested this prediction by studying C57BL/6 mice. This strain bears the *H2* susceptibility haplotype but carries the *Glis3* resistance allele. As shown in Fig. 4, C57BL/6 mice did not show younger onset OA, validating the above model.

### Multi-omic analysis of human datasets

To establish the clinical relevance of these findings in humans and identify potential effector genes for the genome-wide significant QTL on mouse chromosomes 17 and 19, we performed a multi-omic analysis of the homologous regions in the human genome on chromosomes 6p21.32 and 9p24.2, respectively. We first checked publicly available OA GWAS meta-analysis results from a study in 24,955 cases and 378,169 controls [17] for evidence of significant associations at these genomic loci relevant to knee OA. Once confirmed, eQTL and mQTL data from the whole blood [36–38] were combined with the GWAS results to identify potential effector genes and mechanisms driving the associations. This was performed using the SMR software [36], which applies the principles of Mendelian randomisation to identify DNAm sites associated with gene expression and disease through shared (pleiotropic) genetic effects. We first mapped the methylome to the transcriptome to identify the associations between DNAm sites and expression of nearby genes, followed by testing for association between gene expression levels and knee OA, and DNAm sites and knee OA.

**Fig. 5:**
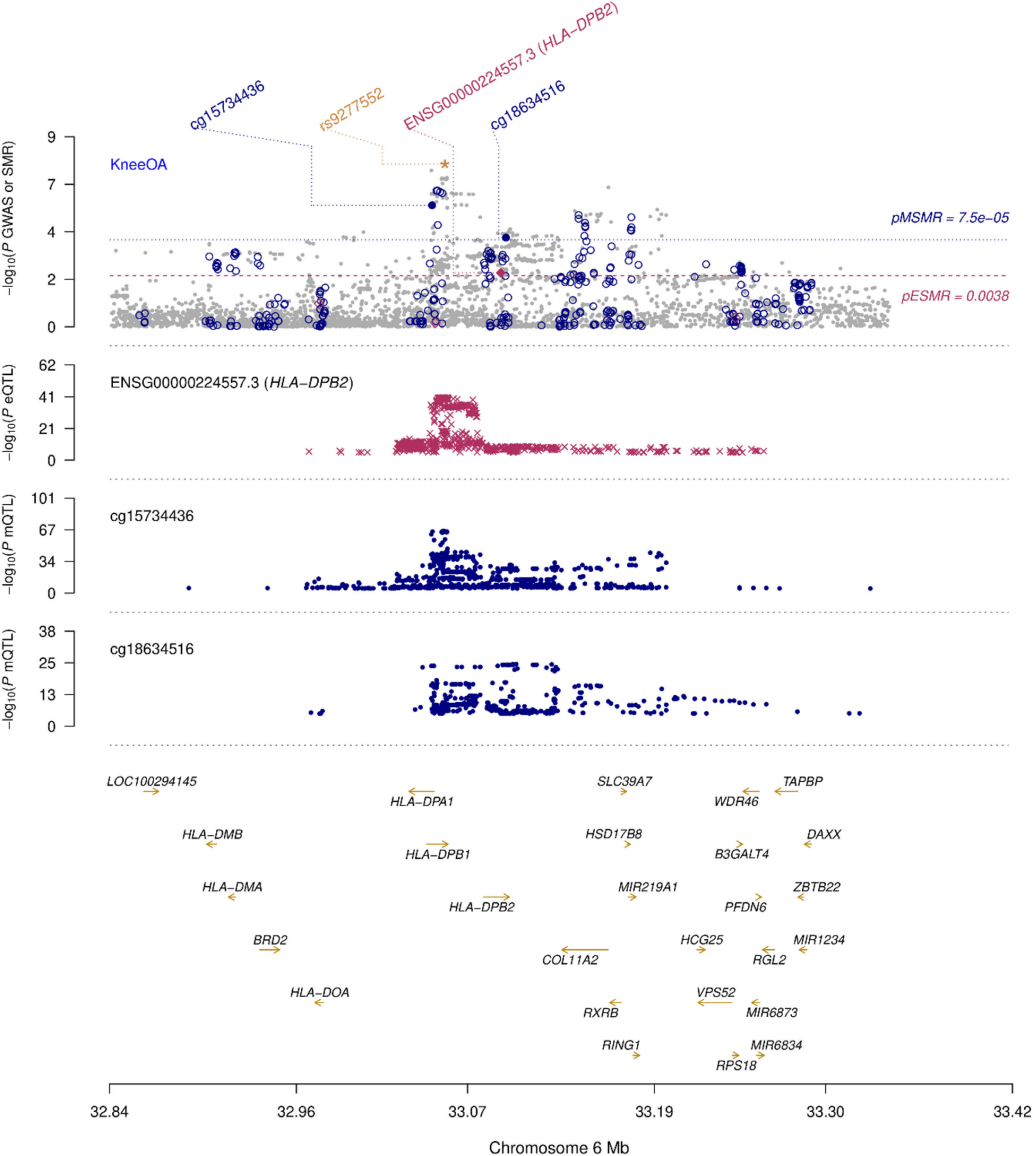
Multi-omics SMR plot of the locus on human chromosome 6p21.32 demonstrating significant pleiotropic associations between the knee OA GWAS, whole-blood eQTL and whole-blood mQTL datasets. Genetic variants are depicted (*x* axis) along with their *P* value (− log_10_) (*y* axis). The upper frame presents the knee OA GWAS association data complete with multiple testing-corrected significance thresholds, location of DNAm probes and lead GWAS variant (rs9277552). The second frame displays the eQTL association data for *HLA-DPB2*, while the third and fourth frames present the mQTL results for the DNAm sites cg15734436 and cg18634516, respectively. Gene positions and direction of transcription are indicated in the lower frame. pMSMR, multiple-testing corrected significance threshold for the mQTL/GWAS SMR analysis; pESMR, multiple testing-corrected significance threshold for the eQTL/GWAS SMR analysis

The 6p21.32 region of the human genome contains a genome-wide significant association signal for knee OA led by the variant *rs9277552* (*P* = 2.0 × 10^−8^), located in the 3′ untranslated region of the *HLA-DPB1* gene. We included all DNAm sites and expressed genes located within ± 500 MB of rs9277552 in the multi-omics SMR analysis. After correction for multiple testing and excluding associations demonstrating *P*_HEIDI_ < 0.01, we identified two DNAm sites, *cg15734436* and *cg18634516*, displaying pleiotropic associations with both knee OA (*P*_SMR_ = 1.8 × 10^−6^ and 6.0 × 10^−5^, respectively) and expression of the nearby pseudogene HLA-DPB2 (*P*_SMR_ = 3.6 × 10^−7^ and 1.7 × 10^−5^, respectively) (Fig. 5). The expression of *HLA-DPB2* was also found to demonstrate pleiotropic association with knee OA after correction for multiple testing (*P*_SMR_ = 2.8 × 10^−3^) (Fig. 5), with increased expression of *HLA-DPB2* associated with a reduced risk of knee OA. Collectively, this suggests that the DNAm sites cg15734436 and cg18634516 are relevant to knee OA through the regulation of *HLA-DPB2* expression.

Locus 9p24.2 contains a genome-wide suggestive association signal for knee OA led by the variant rs3892354 (*P* = 3.3 × 10^−*7*^), located in intron 2 of the *GLIS3* gene. The SMR analysis identified two DNAm sites located within 500 kb of *rs3892354*, *cg13696752* and *cg14514600*, displaying pleiotropic associations with expression of the gene *PPAPDC2* (*P*_SMR_ = 1.2 × 10^−7^ and 1.8 × 10^−5^, respectively). However, neither of these DNAm sites nor the expression of *PPAPDC2* were found to be significantly associated with knee OA (*P*_SMR_ = 0.35–0.93).

## Discussion

This study is the first to use the CC resource to investigate the genetics of spontaneous OA with findings being further characterised using human genetic data. With 43 CC lines and 222 mice, it also provides an increase in power compared with a previous study of bone architecture that used 31 CC lines and a total of 160 mice [24]. We identified two genome-wide significant QTL peaks on chromosomes 19 and 17; these were associated with OA in the total cohort and younger onset cohort, respectively.

By integrating knee OA GWAS results with human data (eQTL and mQTL data from whole-blood), multiple association signals were revealed relevant to the 6p21.32 locus, the human region syntenic to the mouse chromosome 17 QTL. Our results suggested that regulation of *HLA-DPB2* expression by the DNAm sites *cg15734436* and *cg18634516* may have a role in the development of knee OA. There has been emerging evidence to support the notion that there may be an inflammatory component to the pathogenesis of OA, superseding the historical view of OA being a disease caused strictly by mechanical degeneration. A GWAS in a Japanese cohort of 906 individuals with knee OA against 3396 controls identified two SNPs, *rs7775228* and *rs10947262*, as associated with knee OA susceptibility. The identified SNPs reached genome-wide significance within a region containing HLA class II/III genes [43]. These results together with the findings from our study strengthen the argument for an immune role in the pathogenesis of knee OA. It should also be noted that the *COL11A2* gene, which presents very strongly as an OA candidate gene, is also located within the 6p21.32 locus, and that the lead knee OA GWAS variant from this region (*rs9277552*) is in low/moderate LD (*r*^2^ = 0.32) with a missense variant in *COL11A2* (*rs2855430*). Mutation in the human *COL11A2* gene has been found to cause otospondylomegaepiphyseal dysplasia, a disorder characterised by a variety of skeletal abnormalities including young onset OA [44, 45]. However, it should also be noted that *rs9277552* is in strong LD (*r*^2^ > 0.6) with a number of variants related to immune conditions, such as Sjogren’s syndrome [46], granulomatosis with polyangiitis (Wegener’s granulomatosis) [47] and chronic/persistent hepatitis B infection [48–51]. Thus, variation within this locus on human chromosome 6p21.32 seems to have functional effects on the immune system. Significantly, DNA methylation at specific sites in the MHC region seems to affect OA susceptibility in both the CC mice and in humans.

Although our multi-omic analysis did not produce significant findings for human chromosome 9p24.2, it is likely that the genome-wide suggestive association signal for knee OA located within this locus in the human GWAS data is relevant to the *GLIS3* gene. The human *GLIS3* gene has previously been identified as associated with OA by Casalone et al., using GWAS in individuals who underwent hip and/or knee replacements [19]. A recent study has shown that an increase of miR-106a-5p expression in chondrocytes reduced the histological score of OA-induced mice by inhibiting Glis3 production. This study also identified a natural compound that was shown to increase miR-106a-5p expression, which further demonstrates that *GLIS3* is an important drug target for OA [52]. *GLIS3* has been implicated in previous studies investigating diabetes and hypothyroidism which have been deemed risk factors for OA development [53–55]. The association of *Glis3* with OA in the complete cohort of CC mice in our study suggests that it contributes to the overall risk of OA development. The identification of this gene validates our overall approach of using the CC mice to discover the genetic factors that are associated with the development of spontaneous OA.

A recent study published by Steinberg et al. generated eQTL and protein QTL (pQTL) data specific to OA tissues (i.e. primary cartilage and synovium) and performed an integrative analysis with human OA GWAS results to identify potential effector genes [56]. They did not report significant associations between expression of the *HLA-DPB2* gene and knee OA, which demonstrates the utility of our targeted approach to detect significant loci that may be missed in genome-wide analyses. It is also possible that the genetic regulatory effects observed in our study for the *HLA-DPB2* locus are specific to blood, a theory supported by the immune role of this locus. Kreitmaier et al. generated mQTL data for the same OA tissues and reported colocalisation between mQTL signals at the *HLA-DPB2* locus and knee OA GWAS associations, which supports our epigenetic findings for this locus [57]. We considered using these OA tissue -omics datasets in our study, however considering that integrative analyses with OA GWAS data had already been performed in these publications we decided not to repeat the analysis.

The association with *Glis3* and severe OA in the complete cohort was derived from a disease allele inherited from the CAST and AJ founders. Using the suggestive association of the same region in the younger onset cohort in conjunction with the strong association of the H2 locus with the younger onset of OA, we identified several CC strains that carry the *Glis3* allele derived from the C57BL/6 J founder and demonstrate resistance to severe OA despite carrying the H2 risk allele. Since the H2 risk allele in the CC model was also inherited from the C57BL/6 J founder strain, we were able to confirm this finding by demonstrating that C57BL/6 J mice at 8 months did not develop severe OA. This supports the findings of Ji et al. and the importance of *Glis3* as a disease-modifying therapeutic target in OA [52]. This finding can direct further studies towards targeting *Glis3* for individuals with younger OA onset with an elevated risk of revision due to aseptic loosening [58].

The identification of these genetic effects for OA provides a compelling case for further investigation into genetic variants that affect the function of these genes. In addition, drug screens for therapeutic agents which might modulate the signal transduction pathways relevant to these genes to treat or prevent the disease should now be prioritised. Furthermore, these variants should be examined for potential use as diagnostic markers to better assess the risk of OA development and progression in patients. The concept of using SNP markers as a diagnostic tool has been shown previously by Blanco et al., to be significantly more effective than radiographic assessment and age classification used currently [14].

Despite the success in identifying two genes associated with OA, the study has some limitations. The relatively small numbers of individual mice available per strain resulted in an imbalance of sex and age in some cases (Additional file 1: Table S5). This also contributed to a reduction of biological replicates when investigating the sub-population of younger age groups. Ideal circumstances would provide equal numbers of male and female mice across all age groups, but the nature of the CC resource-limited our ability to obtain sufficient mice at desired time points. However, it has been shown that even small cohorts of CC mice can be used to map traits reliably [27]. The CC cohort is a genetically diverse population, capturing over 90% of the common genetic variation of the species, so in this respect is more powerful than human GWAS. Significantly, the CC has been used to identify genes for complex traits that could not be found in human GWAS analyses of thousands of individuals [28]. The validation of mouse QTL in highly powered human OA GWAS further shows that the CC can identify genetic loci associated with OA and other complex diseases.

In conclusion, we identified two genetic loci associated with the development of spontaneous knee OA in mice. Multi-omic analysis of human knee OA GWAS, whole-blood eQTL and mQTL data suggests that regulation of *HLA-DPB2* expression by DNAm sites may have a role in the development of knee OA, highlighting a potential immune role in the disease. Our data also supports recent discoveries made in large human OA GWAS, confirming the value of mouse models of OA in the greater understanding of human OA. Further investigation into the variations in these genes and their functions may provide new diagnostic tools and potential treatment targets for knee OA.

### Supplementary Information

**Additional file 1: Table S1.** Grades of OA as determined by the OARSI grading system. **Table S2.** Risk and resistance alleles for OA in the 8 – 12 month cohort. **Table S3.** CAST-specific missense SNPs on Chromosome 19 between 27.82 and 31.51 Mbp. **Table S4.** CAST-specific SNPs on Chromosome 17 between 35 and 38.8 Mbp. **Table S5.** Strain sample numbers.

**Additional file 2. **Supplementary Methods.

## Data Availability

Data are available upon reasonable request.

## Abbreviations

ACL: Anterior cruciate ligament
CC: Collaborative Cross
DOQTL R: Diversity Outbred Quantitative Trait Locus mapping pipeline
eQTL: Expression quantitative trait locus
GWAS: Genome-wide association study
HEIDI: Heterogeneity in dependent instruments test
HLA: Human leukocyte antigens
LD: Linkage disequilibrium
LFC: Lateral femoral condyle
LOD: Logarithm of the odds
LTP: Lateral tibial plateau
MFC: Medial femoral condyle
MHC: Major histocompatibility complex
mQTL: Methylation quantitative trait locus
MTP: Medial tibial plateau
OA: Osteoarthritis
OARSI: Osteoarthritis Research Society International
PCL: Posterior cruciate ligament
QTL: Quantitative trait locus
RI: Recombinant inbred
SMR: Summary-based Mendelian randomisation
SNP: Single nucleotide polymorphism
