# Supplementary material for: Age-dependent genetic regulation of osteoarthritis: independent effects of immune system genes
Source: Arthritis Res Ther. 2023 Dec 1;25:232. doi: 10.1186/s13075-023-03216-2 (PMC10691153; doi:10.1186/s13075-023-03216-2)
Supplement: Supplementary file 1 — Additional file 1: Table S1. Grades of OA as determined by the OARSI grading system. Table S2. Risk and resistance alleles for OA in the 8 – 12 month cohort. Table S3. CAST-specific missense SNPs on Chromosome 19 between 27.82 and 31.51 Mbp. Table S4. CAST-specific SNPs on Chromosome 17 between 35 and 38.8 Mbp. Table S5. Strain sample numbers. [file 13075_2023_3216_MOESM1_ESM.docx]

| **Score** | **Observation** |
| --- | --- |
| 0 | Normal |
| 1 | Small fibrillations without loss of cartilage (loss of Safranin O staining without structural changes considered a score of 0.5) |
| 2 | Vertical clefts down to the layer immediately below the superficial layer and some loss of surface lamina |
| 3 | Vertical clefts/erosion to the calcified cartilage extending to <25% of the articular surface |
| 4 | Vertical clefts/erosion to the calcified cartilage extending to 25-50% of the articular surface |
| 5 | Vertical clefts/erosion to the calcified cartilage extending to 50-75% of the articular surface |
| 6 | Vertical clefts/erosion to the calcified cartilage extending to >75% of the articular surface |

**Supplementary Table 1. Grades of OA as determined by the OARSI grading system**

| Early onset cohort | OA score (Median) | Risk both loci | Risk allele *Glis3* | Resistant allele *Glis3* | Risk allele H2 | Risk allele H2/Resistant allele *Glis3* | No susceptibility risk |
| --- | --- | --- | --- | --- | --- | --- | --- |
| BOON | 0 |  |  |  |  |  |  |
| DET3 | 0 |  |  |  |  |  |  |
| GAV | 0 |  |  |  |  |  |  |
| MOP | 0 |  |  |  |  |  |  |
| PER2 | 0 |  |  |  |  |  |  |
| PIPING | 0 |  |  |  |  |  |  |
| SAT | 0 |  |  |  |  |  |  |
| TOFU | 0 |  |  |  |  |  |  |
| VIT | 0 |  |  |  |  |  |  |
| WOB2 | 0 |  |  |  |  |  |  |
| ZIF2 | 0 |  |  |  |  |  |  |
| YID | 0.5 |  |  |  |  |  |  |
| BEM | 1 |  |  |  |  |  |  |
| WAD | 1 |  |  |  |  |  |  |
| CAMERON | 1 |  |  |  |  |  |  |
| GEK2 | 1 |  |  |  |  |  |  |
| HAX2 | 1 |  |  |  |  |  |  |
| HAZ | 1 |  |  |  |  |  |  |
| STUCKY | 1 |  |  |  |  |  |  |
| HIP | 2.5 |  |  |  |  |  |  |
| PEF | 3.5 |  |  |  |  |  |  |
| POH | 4 |  |  |  |  |  |  |
| JUD | 4 |  |  |  |  |  |  |
| JEUNE | 6.5 |  |  |  |  |  |  |

**Supplementary Table 2. Risk and resistance alleles for OA in the 8 – 12 month cohort**

| Gene | Chr | bp | SNP | B6 | 129 | AJ | CAST | NOD | NZO | PWK | WSB | CAST UNIQUE? |  |
| --- | --- | --- | --- | --- | --- | --- | --- | --- | --- | --- | --- | --- | --- |
| *Glis3* | 19 | 28,337,223 | [rs245241207](https://aus01.safelinks.protection.outlook.com/?url=http%3A%2F%2Fwww.ncbi.nlm.nih.gov%2Fprojects%2FSNP%2Fsnp_ref.cgi%3Frs%3Drs245241207&data=04%7C01%7Cjacob.kenny%40uwa.edu.au%7C146f55219dd742511f7b08d93787d1cb%7C05894af0cb2846d8871674cdb46e2226%7C1%7C0%7C637601878748043572%7CUnknown%7CTWFpbGZsb3d8eyJWIjoiMC4wLjAwMDAiLCJQIjoiV2luMzIiLCJBTiI6Ik1haWwiLCJXVCI6Mn0%3D%7C1000&sdata=u3r5JUOz1WmEtxUDDdTMTfBrgsWjFIeqedYeDTon5Qg%3D&reserved=0) | T | - | - | A | - | - | - | - | 1 |  |
| *Glis3* | 19 | 28,337,279 | [rs215116170](https://aus01.safelinks.protection.outlook.com/?url=http%3A%2F%2Fwww.ncbi.nlm.nih.gov%2Fprojects%2FSNP%2Fsnp_ref.cgi%3Frs%3Drs215116170&data=04%7C01%7Cjacob.kenny%40uwa.edu.au%7C146f55219dd742511f7b08d93787d1cb%7C05894af0cb2846d8871674cdb46e2226%7C1%7C0%7C637601878748053530%7CUnknown%7CTWFpbGZsb3d8eyJWIjoiMC4wLjAwMDAiLCJQIjoiV2luMzIiLCJBTiI6Ik1haWwiLCJXVCI6Mn0%3D%7C1000&sdata=a65G9xDzqLJAYZJ%2FB0cSdBn2b1Hj%2BynjwqvyvaKgjR4%3D&reserved=0) | A | - | - | T | - | - | - | - | 1 |  |
| *Glis3* | 19 | 28,337,316 | [rs232469133](https://aus01.safelinks.protection.outlook.com/?url=http%3A%2F%2Fwww.ncbi.nlm.nih.gov%2Fprojects%2FSNP%2Fsnp_ref.cgi%3Frs%3Drs232469133&data=04%7C01%7Cjacob.kenny%40uwa.edu.au%7C146f55219dd742511f7b08d93787d1cb%7C05894af0cb2846d8871674cdb46e2226%7C1%7C0%7C637601878748053530%7CUnknown%7CTWFpbGZsb3d8eyJWIjoiMC4wLjAwMDAiLCJQIjoiV2luMzIiLCJBTiI6Ik1haWwiLCJXVCI6Mn0%3D%7C1000&sdata=xifuLWELbkbK8Rk296Ubp0srvrEVUnbV%2FQ7wCZvmGMo%3D&reserved=0) | C | - | - | T | - | - | - | - | 1 |  |
| *Glis3* | 19 | 28,605,937 | [rs30347506](https://aus01.safelinks.protection.outlook.com/?url=http%3A%2F%2Fwww.ncbi.nlm.nih.gov%2Fprojects%2FSNP%2Fsnp_ref.cgi%3Frs%3Drs30347506&data=04%7C01%7Cjacob.kenny%40uwa.edu.au%7C146f55219dd742511f7b08d93787d1cb%7C05894af0cb2846d8871674cdb46e2226%7C1%7C0%7C637601878748063484%7CUnknown%7CTWFpbGZsb3d8eyJWIjoiMC4wLjAwMDAiLCJQIjoiV2luMzIiLCJBTiI6Ik1haWwiLCJXVCI6Mn0%3D%7C1000&sdata=Z93D9TUEdcywouKcKaUwuLBGA8GtioKXc8xfIpunnsM%3D&reserved=0) | C | - | T | - | - | - | - | T |  |  |
| *Glis3* | 19 | 28,605,956 | [rs51654120](https://aus01.safelinks.protection.outlook.com/?url=http%3A%2F%2Fwww.ncbi.nlm.nih.gov%2Fprojects%2FSNP%2Fsnp_ref.cgi%3Frs%3Drs51654120&data=04%7C01%7Cjacob.kenny%40uwa.edu.au%7C146f55219dd742511f7b08d93787d1cb%7C05894af0cb2846d8871674cdb46e2226%7C1%7C0%7C637601878748063484%7CUnknown%7CTWFpbGZsb3d8eyJWIjoiMC4wLjAwMDAiLCJQIjoiV2luMzIiLCJBTiI6Ik1haWwiLCJXVCI6Mn0%3D%7C1000&sdata=Koe5b2GTfc6UI2CDAguHgg4q8XHomulrtrK2wJekiT4%3D&reserved=0) | T | - | - | C | - | - | ~ | - | 1 |  |
| *Glis3* | 19 | 28,740,524 | [rs246164195](https://aus01.safelinks.protection.outlook.com/?url=http%3A%2F%2Fwww.ncbi.nlm.nih.gov%2Fprojects%2FSNP%2Fsnp_ref.cgi%3Frs%3Drs246164195&data=04%7C01%7Cjacob.kenny%40uwa.edu.au%7C146f55219dd742511f7b08d93787d1cb%7C05894af0cb2846d8871674cdb46e2226%7C1%7C0%7C637601878748073442%7CUnknown%7CTWFpbGZsb3d8eyJWIjoiMC4wLjAwMDAiLCJQIjoiV2luMzIiLCJBTiI6Ik1haWwiLCJXVCI6Mn0%3D%7C1000&sdata=CXMtxCsSiwKsFx4nhFP%2BHLZAIFThqHGnmv%2BLP0ntzmI%3D&reserved=0) | C | - | - | G | - | - | - | - | 1 |  |
| *Glis3* | 19 | 28,740,528 | - | C | - | - | T | - | - | - | - | 1 |  |
| *Rps15a-ps2* | 19 | 28,816,909 | [rs243578915](https://aus01.safelinks.protection.outlook.com/?url=http%3A%2F%2Fwww.ncbi.nlm.nih.gov%2Fprojects%2FSNP%2Fsnp_ref.cgi%3Frs%3Drs243578915&data=04%7C01%7Cjacob.kenny%40uwa.edu.au%7C146f55219dd742511f7b08d93787d1cb%7C05894af0cb2846d8871674cdb46e2226%7C1%7C0%7C637601878748073442%7CUnknown%7CTWFpbGZsb3d8eyJWIjoiMC4wLjAwMDAiLCJQIjoiV2luMzIiLCJBTiI6Ik1haWwiLCJXVCI6Mn0%3D%7C1000&sdata=%2F7vombosszDQNSnmlF0EI9SVSCLFTwMcRvh6McSkg%2FU%3D&reserved=0) | C | - | - | T | - | - | - | - | 1 |  |
| *4430402I18Rik* | 19 | 29,018,919 | [rs47287241](https://aus01.safelinks.protection.outlook.com/?url=http%3A%2F%2Fwww.ncbi.nlm.nih.gov%2Fprojects%2FSNP%2Fsnp_ref.cgi%3Frs%3Drs47287241&data=04%7C01%7Cjacob.kenny%40uwa.edu.au%7C146f55219dd742511f7b08d93787d1cb%7C05894af0cb2846d8871674cdb46e2226%7C1%7C0%7C637601878748083398%7CUnknown%7CTWFpbGZsb3d8eyJWIjoiMC4wLjAwMDAiLCJQIjoiV2luMzIiLCJBTiI6Ik1haWwiLCJXVCI6Mn0%3D%7C1000&sdata=UVTMCHpuaJZ8kBLfHXaDhidIPfgUnJvGwnmh2N2x7CU%3D&reserved=0) | G | - | - | A | - | - | - | - | 1 |  |
| *Pdcd1lg2* | 19 | 29,529,066 | [rs236361179](https://aus01.safelinks.protection.outlook.com/?url=http%3A%2F%2Fwww.ncbi.nlm.nih.gov%2Fprojects%2FSNP%2Fsnp_ref.cgi%3Frs%3Drs236361179&data=04%7C01%7Cjacob.kenny%40uwa.edu.au%7C146f55219dd742511f7b08d93787d1cb%7C05894af0cb2846d8871674cdb46e2226%7C1%7C0%7C637601878748083398%7CUnknown%7CTWFpbGZsb3d8eyJWIjoiMC4wLjAwMDAiLCJQIjoiV2luMzIiLCJBTiI6Ik1haWwiLCJXVCI6Mn0%3D%7C1000&sdata=8jNiCY7C9HdOMvH6%2FYnmm7F4Vh2e%2FSjmVaqHMez70o8%3D&reserved=0) | A | - | - | G | - | - | - | - | 1 |  |
| *C030046E11Rik* | 19 | 29,607,719 | [rs37776272](https://aus01.safelinks.protection.outlook.com/?url=http%3A%2F%2Fwww.ncbi.nlm.nih.gov%2Fprojects%2FSNP%2Fsnp_ref.cgi%3Frs%3Drs37776272&data=04%7C01%7Cjacob.kenny%40uwa.edu.au%7C146f55219dd742511f7b08d93787d1cb%7C05894af0cb2846d8871674cdb46e2226%7C1%7C0%7C637601878748093352%7CUnknown%7CTWFpbGZsb3d8eyJWIjoiMC4wLjAwMDAiLCJQIjoiV2luMzIiLCJBTiI6Ik1haWwiLCJXVCI6Mn0%3D%7C1000&sdata=pLWnoWXSrQnnIo7qp8SjELU1joYEG4epSPihZfOzDsM%3D&reserved=0) | A | - | - | G | - | - | - | - | 1 |  |
| *C030046E11Rik* | 19 | 29,636,531 | [rs36650535](https://aus01.safelinks.protection.outlook.com/?url=http%3A%2F%2Fwww.ncbi.nlm.nih.gov%2Fprojects%2FSNP%2Fsnp_ref.cgi%3Frs%3Drs36650535&data=04%7C01%7Cjacob.kenny%40uwa.edu.au%7C146f55219dd742511f7b08d93787d1cb%7C05894af0cb2846d8871674cdb46e2226%7C1%7C0%7C637601878748093352%7CUnknown%7CTWFpbGZsb3d8eyJWIjoiMC4wLjAwMDAiLCJQIjoiV2luMzIiLCJBTiI6Ik1haWwiLCJXVCI6Mn0%3D%7C1000&sdata=kL20glqjirrGo%2FPElShOEWnRN1zZlhUNQFP78429hyA%3D&reserved=0) | C | - | - | T | - | - | - | - | 1 |  |
| *Mlana* | 19 | 29,774,596 | [rs217069336](https://aus01.safelinks.protection.outlook.com/?url=http%3A%2F%2Fwww.ncbi.nlm.nih.gov%2Fprojects%2FSNP%2Fsnp_ref.cgi%3Frs%3Drs217069336&data=04%7C01%7Cjacob.kenny%40uwa.edu.au%7C146f55219dd742511f7b08d93787d1cb%7C05894af0cb2846d8871674cdb46e2226%7C1%7C0%7C637601878748103310%7CUnknown%7CTWFpbGZsb3d8eyJWIjoiMC4wLjAwMDAiLCJQIjoiV2luMzIiLCJBTiI6Ik1haWwiLCJXVCI6Mn0%3D%7C1000&sdata=Kpee8kvLJqczYcBmCRenFdRYvrRs8AqB5iA2frIN3A8%3D&reserved=0) | C | - | - | G | - | - | - | - | 1 |  |
| *Mlana* | 19 | 29,774,637 | [rs257938916](https://aus01.safelinks.protection.outlook.com/?url=http%3A%2F%2Fwww.ncbi.nlm.nih.gov%2Fprojects%2FSNP%2Fsnp_ref.cgi%3Frs%3Drs257938916&data=04%7C01%7Cjacob.kenny%40uwa.edu.au%7C146f55219dd742511f7b08d93787d1cb%7C05894af0cb2846d8871674cdb46e2226%7C1%7C0%7C637601878748103310%7CUnknown%7CTWFpbGZsb3d8eyJWIjoiMC4wLjAwMDAiLCJQIjoiV2luMzIiLCJBTiI6Ik1haWwiLCJXVCI6Mn0%3D%7C1000&sdata=qfSiW7VPdBcGDw1wsQt88W3JtLTLjgjXuEGFoUtAS8Y%3D&reserved=0) | A | - | - | C | - | - | - | - | 1 |  |
| *Mlana* | 19 | 29,774,651 | [rs215582999](https://aus01.safelinks.protection.outlook.com/?url=http%3A%2F%2Fwww.ncbi.nlm.nih.gov%2Fprojects%2FSNP%2Fsnp_ref.cgi%3Frs%3Drs215582999&data=04%7C01%7Cjacob.kenny%40uwa.edu.au%7C146f55219dd742511f7b08d93787d1cb%7C05894af0cb2846d8871674cdb46e2226%7C1%7C0%7C637601878748113267%7CUnknown%7CTWFpbGZsb3d8eyJWIjoiMC4wLjAwMDAiLCJQIjoiV2luMzIiLCJBTiI6Ik1haWwiLCJXVCI6Mn0%3D%7C1000&sdata=rulC4F84Mwsh%2FmwDLymKfalmiL%2Fe88F0HXRhmqcNL64%3D&reserved=0) | C | - | - | T | - | - | - | - | 1 |  |
| *Mlana* | 19 | 29,781,308 | [rs225407020](https://aus01.safelinks.protection.outlook.com/?url=http%3A%2F%2Fwww.ncbi.nlm.nih.gov%2Fprojects%2FSNP%2Fsnp_ref.cgi%3Frs%3Drs225407020&data=04%7C01%7Cjacob.kenny%40uwa.edu.au%7C146f55219dd742511f7b08d93787d1cb%7C05894af0cb2846d8871674cdb46e2226%7C1%7C0%7C637601878748113267%7CUnknown%7CTWFpbGZsb3d8eyJWIjoiMC4wLjAwMDAiLCJQIjoiV2luMzIiLCJBTiI6Ik1haWwiLCJXVCI6Mn0%3D%7C1000&sdata=fT6FwOOg4GFQbxqwsD7Wkuziv%2BahRw8M8GYUIVaIxEI%3D&reserved=0) | G | - | - | A | - | - | - | - | 1 |  |
| *Mlana* | 19 | 29,781,314 | [rs241111360](https://aus01.safelinks.protection.outlook.com/?url=http%3A%2F%2Fwww.ncbi.nlm.nih.gov%2Fprojects%2FSNP%2Fsnp_ref.cgi%3Frs%3Drs241111360&data=04%7C01%7Cjacob.kenny%40uwa.edu.au%7C146f55219dd742511f7b08d93787d1cb%7C05894af0cb2846d8871674cdb46e2226%7C1%7C0%7C637601878748123223%7CUnknown%7CTWFpbGZsb3d8eyJWIjoiMC4wLjAwMDAiLCJQIjoiV2luMzIiLCJBTiI6Ik1haWwiLCJXVCI6Mn0%3D%7C1000&sdata=JaWelQPdFo0ZVDN5Jcd4tZWLM%2F43wi5QppeR3HjwJps%3D&reserved=0) | T | - | - | C | - | - | - | - | 1 |  |
| *Mlana* | 19 | 29,781,407 | [rs238735309](https://aus01.safelinks.protection.outlook.com/?url=http%3A%2F%2Fwww.ncbi.nlm.nih.gov%2Fprojects%2FSNP%2Fsnp_ref.cgi%3Frs%3Drs238735309&data=04%7C01%7Cjacob.kenny%40uwa.edu.au%7C146f55219dd742511f7b08d93787d1cb%7C05894af0cb2846d8871674cdb46e2226%7C1%7C0%7C637601878748123223%7CUnknown%7CTWFpbGZsb3d8eyJWIjoiMC4wLjAwMDAiLCJQIjoiV2luMzIiLCJBTiI6Ik1haWwiLCJXVCI6Mn0%3D%7C1000&sdata=jk6pOi8GRlTubr3yYYQUzuKO9T0pTD7odb4PINB4zKs%3D&reserved=0) | C | - | - | T | - | - | - | - | 1 |  |
| *9930021J03Rik* | 19 | 29,791,090 | [rs222820358](https://aus01.safelinks.protection.outlook.com/?url=http%3A%2F%2Fwww.ncbi.nlm.nih.gov%2Fprojects%2FSNP%2Fsnp_ref.cgi%3Frs%3Drs222820358&data=04%7C01%7Cjacob.kenny%40uwa.edu.au%7C146f55219dd742511f7b08d93787d1cb%7C05894af0cb2846d8871674cdb46e2226%7C1%7C0%7C637601878748133182%7CUnknown%7CTWFpbGZsb3d8eyJWIjoiMC4wLjAwMDAiLCJQIjoiV2luMzIiLCJBTiI6Ik1haWwiLCJXVCI6Mn0%3D%7C1000&sdata=5DPD5KmrBZUBMBybkrME48Pj8ReuxG2U9CynH8TYZBo%3D&reserved=0) | G | - | - | A | - | - | - | - | 1 |  |
| *9930021J03Rik* | 19 | 29,793,391 | [rs36444910](https://aus01.safelinks.protection.outlook.com/?url=http%3A%2F%2Fwww.ncbi.nlm.nih.gov%2Fprojects%2FSNP%2Fsnp_ref.cgi%3Frs%3Drs36444910&data=04%7C01%7Cjacob.kenny%40uwa.edu.au%7C146f55219dd742511f7b08d93787d1cb%7C05894af0cb2846d8871674cdb46e2226%7C1%7C0%7C637601878748143136%7CUnknown%7CTWFpbGZsb3d8eyJWIjoiMC4wLjAwMDAiLCJQIjoiV2luMzIiLCJBTiI6Ik1haWwiLCJXVCI6Mn0%3D%7C1000&sdata=1x5NjLPJJgzdmiTbNTfxs5tSsgklHl6GXId91nJz8xM%3D&reserved=0) | C | - | - | A | - | - | - | - | 1 |  |
| *9930021J03Rik* | 19 | 29,795,140 | [rs37597785](https://aus01.safelinks.protection.outlook.com/?url=http%3A%2F%2Fwww.ncbi.nlm.nih.gov%2Fprojects%2FSNP%2Fsnp_ref.cgi%3Frs%3Drs37597785&data=04%7C01%7Cjacob.kenny%40uwa.edu.au%7C146f55219dd742511f7b08d93787d1cb%7C05894af0cb2846d8871674cdb46e2226%7C1%7C0%7C637601878748143136%7CUnknown%7CTWFpbGZsb3d8eyJWIjoiMC4wLjAwMDAiLCJQIjoiV2luMzIiLCJBTiI6Ik1haWwiLCJXVCI6Mn0%3D%7C1000&sdata=%2FDleY1FHd9PfsbiLMe2X%2BMBn4%2BG4Zwp6IrFXZ04lWc4%3D&reserved=0) | C | - | - | A | - | - | - | - | 1 |  |
| *Rpl31-ps20* | 19 | 30,235,119 | [rs221895768](https://aus01.safelinks.protection.outlook.com/?url=http%3A%2F%2Fwww.ncbi.nlm.nih.gov%2Fprojects%2FSNP%2Fsnp_ref.cgi%3Frs%3Drs221895768&data=04%7C01%7Cjacob.kenny%40uwa.edu.au%7C146f55219dd742511f7b08d93787d1cb%7C05894af0cb2846d8871674cdb46e2226%7C1%7C0%7C637601878748153092%7CUnknown%7CTWFpbGZsb3d8eyJWIjoiMC4wLjAwMDAiLCJQIjoiV2luMzIiLCJBTiI6Ik1haWwiLCJXVCI6Mn0%3D%7C1000&sdata=WlU1NjjFlH2qk%2B0PtXPIpqbEY3WMuILvdkIw1nqoZRs%3D&reserved=0) | A | - | - | G | - | - | - | - | 1 |  |
| *Rpl31-ps20* | 19 | 30,235,149 | - | A | - | - | C | - | - | - | - | 1 |  |
| *Gldc* | 19 | 30,248,954 | [rs244793142](https://aus01.safelinks.protection.outlook.com/?url=http%3A%2F%2Fwww.ncbi.nlm.nih.gov%2Fprojects%2FSNP%2Fsnp_ref.cgi%3Frs%3Drs244793142&data=04%7C01%7Cjacob.kenny%40uwa.edu.au%7C146f55219dd742511f7b08d93787d1cb%7C05894af0cb2846d8871674cdb46e2226%7C1%7C0%7C637601878748153092%7CUnknown%7CTWFpbGZsb3d8eyJWIjoiMC4wLjAwMDAiLCJQIjoiV2luMzIiLCJBTiI6Ik1haWwiLCJXVCI6Mn0%3D%7C1000&sdata=CNODv6O3lpelQJIu%2Bq8OZU6XozE6gU%2F5G88qqgg1XkU%3D&reserved=0) | G | - | - | A | - | - | - | - | 1 |  |
| *Mbl2* | 19 | 30,313,970 | [rs231405964](https://aus01.safelinks.protection.outlook.com/?url=http%3A%2F%2Fwww.ncbi.nlm.nih.gov%2Fprojects%2FSNP%2Fsnp_ref.cgi%3Frs%3Drs231405964&data=04%7C01%7Cjacob.kenny%40uwa.edu.au%7C146f55219dd742511f7b08d93787d1cb%7C05894af0cb2846d8871674cdb46e2226%7C1%7C0%7C637601878748163047%7CUnknown%7CTWFpbGZsb3d8eyJWIjoiMC4wLjAwMDAiLCJQIjoiV2luMzIiLCJBTiI6Ik1haWwiLCJXVCI6Mn0%3D%7C1000&sdata=D3DTJnhpN4OZvtiV1rQultx2dS6J8Nj5OO7FTsZwDPY%3D&reserved=0) | C | - | - | T | - | - | - | - | 1 |  |
| *Dkk1* | 19 | 30,621,914 | [rs36488595](https://aus01.safelinks.protection.outlook.com/?url=http%3A%2F%2Fwww.ncbi.nlm.nih.gov%2Fprojects%2FSNP%2Fsnp_ref.cgi%3Frs%3Drs36488595&data=04%7C01%7Cjacob.kenny%40uwa.edu.au%7C146f55219dd742511f7b08d93787d1cb%7C05894af0cb2846d8871674cdb46e2226%7C1%7C0%7C637601878748163047%7CUnknown%7CTWFpbGZsb3d8eyJWIjoiMC4wLjAwMDAiLCJQIjoiV2luMzIiLCJBTiI6Ik1haWwiLCJXVCI6Mn0%3D%7C1000&sdata=pzOg1UkFfqClUO2DHPPEl7lFNrT%2B8qw9D8KzucRBdQM%3D&reserved=0) | C | - | - | T | - | - | - | - | 1 |  |

**Supplementary Table 3. CAST-specific missense SNPs on Chromosome 19 between 27.82 and 31.51 Mbp**

**Supplementary Table 4. CAST-specific SNPs on Chromosome 17 between 35 and 38.8 Mbp**

| **Gene** | **dbSNP** | | Chr 17 bp | **ENCODE DB** | **SO Term** | **B6** | **129s** | **CAST** | **A/J** | **NOD** | **NZO** | **PWK** | **WSB** |
| --- | --- | --- | --- | --- | --- | --- | --- | --- | --- | --- | --- | --- | --- |
| H2-Q5 | [rs46262027](http://www.ncbi.nlm.nih.gov/projects/SNP/snp_ref.cgi?rs=rs46262027) | | 35529088 | RE | H3K27_trimethylation_site | T | - |  | A | A | A | A | A |
| Gm8801 | [rs107971401^*^](http://www.ncbi.nlm.nih.gov/projects/SNP/snp_ref.cgi?rs=rs107971401) | | 36084381 | Motif | ESR1 | T | C | - | T | T | - | T | T |
| Gm8835 | | [.](http://www.ncbi.nlm.nih.gov/projects/SNP/snp_ref.cgi?rs=.) | 36335276 | RegF | CTCF_binding_site | A |  |  | G | G | G | G | G |
| Gm5682 | | [.](http://www.ncbi.nlm.nih.gov/projects/SNP/snp_ref.cgi?rs=.) | 36347672 | RE | H3K27_trimethylation_site | T |  | - | A | A | A | A | A |
| Gm20391 | | [.](http://www.ncbi.nlm.nih.gov/projects/SNP/snp_ref.cgi?rs=.) | 36374205 | RE | H3K27_trimethylation_site | A | - | - | T | T | T | T | T |
| Trim39 | | [.](http://www.ncbi.nlm.nih.gov/projects/SNP/snp_ref.cgi?rs=.) | 36402659 | RE | H3K36_trimethylation_site | C | - | - | T | T | T | T | T |
| Trim39 | | [.](http://www.ncbi.nlm.nih.gov/projects/SNP/snp_ref.cgi?rs=.) | 36410593 | RE | H3K4_monomethylation site | C | - | - | G | G | G | G | G |
| Gm20454 | | [.](http://www.ncbi.nlm.nih.gov/projects/SNP/snp_ref.cgi?rs=.) | 36666611 | RE | H3K27_trimethylation_site | C | - | - | T | T | T | T | T |
| Gm20525 | | [.](http://www.ncbi.nlm.nih.gov/projects/SNP/snp_ref.cgi?rs=.) | 36855595 | RE | H3K27_trimethylation_site | C |  | - | G | G | G | G | G |
| Trim26 | | [.](http://www.ncbi.nlm.nih.gov/projects/SNP/snp_ref.cgi?rs=.) | 36985146 | RE | H3K36_trimethylation_site | C | - | - | T | T | T | T | T |
| Trim26 | | [.](http://www.ncbi.nlm.nih.gov/projects/SNP/snp_ref.cgi?rs=.) | 36985150 | RE | H3K36_trimethylation_site | G | - | - | C | C | C | C | C |
| Trim26 | | [.](http://www.ncbi.nlm.nih.gov/projects/SNP/snp_ref.cgi?rs=.) | 36985166 | RE | H3K36_trimethylation_site | T | - | - | C | C | C | C | C |
| Trim26 | | [.](http://www.ncbi.nlm.nih.gov/projects/SNP/snp_ref.cgi?rs=.) | 36987143 | RE | H3K36_trimethylation_site | A | - | - | G | G | G | G | G |
| Trim26 | | [.](http://www.ncbi.nlm.nih.gov/projects/SNP/snp_ref.cgi?rs=.) | 36987860 | RE | H3K36_trimethylation_site | G | - | - | A | A | A | A | A |
| Trim26 | | [.](http://www.ncbi.nlm.nih.gov/projects/SNP/snp_ref.cgi?rs=.) | 36989130 | RE | H3K36_trimethylation_site | T | - | - | C | C | C | C | C |
| Trim26 | | [.](http://www.ncbi.nlm.nih.gov/projects/SNP/snp_ref.cgi?rs=.) | 36991436 | RE | H3K36_trimethylation_site | G | - | - | T | T | T | T | T |
| Trim26 | | [.](http://www.ncbi.nlm.nih.gov/projects/SNP/snp_ref.cgi?rs=.) | 36991675 | RE | H3K36_trimethylation_site | A | - | - | G | G | G | G | G |
| Trim15 | | [.](http://www.ncbi.nlm.nih.gov/projects/SNP/snp_ref.cgi?rs=.) | 36994030 | RE | H3K36_trimethylation_site | C | - | - | T | T | T | T | T |
| Trim15 | | [.](http://www.ncbi.nlm.nih.gov/projects/SNP/snp_ref.cgi?rs=.) | 36994898 | RE | H3K36_trimethylation_site | T | - | - | G | G | G | G | G |
| Trim15 | | [.](http://www.ncbi.nlm.nih.gov/projects/SNP/snp_ref.cgi?rs=.) | 36995447 | RE | H3K36_trimethylation_site | C | - | - | A | A | A | G | A |
| Trim15 | | [.](http://www.ncbi.nlm.nih.gov/projects/SNP/snp_ref.cgi?rs=.) | 36995569 | RE | H3K36_trimethylation_site | T | - | - | C | C | C | C | C |
| Trim15 | | [.](http://www.ncbi.nlm.nih.gov/projects/SNP/snp_ref.cgi?rs=.) | 36996563 | RE | H3K36_trimethylation_site | G | - | - | A | A | A | A | A |
| Trim15 | | [.](http://www.ncbi.nlm.nih.gov/projects/SNP/snp_ref.cgi?rs=.) | 36997211 | RE | H3K36_trimethylation_site | T | - | - | C | C | C | C | C |
| Trim15 | | [.](http://www.ncbi.nlm.nih.gov/projects/SNP/snp_ref.cgi?rs=.) | 36997270 | RE | H3K36_trimethylation_site | A | - | - | G | G | G | G | G |
| Trim15 | | [.](http://www.ncbi.nlm.nih.gov/projects/SNP/snp_ref.cgi?rs=.) | 36997280 | RE | H3K36_trimethylation_site | G | - | - | A | A | A | A | A |
| Trim15 | | [.](http://www.ncbi.nlm.nih.gov/projects/SNP/snp_ref.cgi?rs=.) | 36997349 | RE | H3K36_trimethylation_site | G | - | - | A | A | A | A | A |
| Trim15 | | [.](http://www.ncbi.nlm.nih.gov/projects/SNP/snp_ref.cgi?rs=.) | 36997661 | RE | H3K36_trimethylation_site | T | - | - | G | G | G | G | G |
| Trim15 | | [.](http://www.ncbi.nlm.nih.gov/projects/SNP/snp_ref.cgi?rs=.) | 36997669 | RE | H3K36_trimethylation_site | G | - | - | A | A | A | A | A |
| Trim15 | | [.](http://www.ncbi.nlm.nih.gov/projects/SNP/snp_ref.cgi?rs=.) | 36997699 | RE | H3K36_trimethylation_site | A | - | - | G | G | G | G | G |
| Trim15 | | [.](http://www.ncbi.nlm.nih.gov/projects/SNP/snp_ref.cgi?rs=.) | 36997706 | RE | H3K36_trimethylation_site | A | - | - | G | G | G | G | G |
| Trim15 | | [.](http://www.ncbi.nlm.nih.gov/projects/SNP/snp_ref.cgi?rs=.) | 36997959 | RE | H3K36_trimethylation_site | A | - | - | G | G | G | G | G |
| Trim15 | | [.](http://www.ncbi.nlm.nih.gov/projects/SNP/snp_ref.cgi?rs=.) | 36998145 | RE | H3K36_trimethylation_site | A | - | - | T | T | T | T | T |
| Trim15 | | [.](http://www.ncbi.nlm.nih.gov/projects/SNP/snp_ref.cgi?rs=.) | 36998198 | RegF | CTCF_binding_site | G | - | - | A | A | A | A | A |
| Trim15 | | [.](http://www.ncbi.nlm.nih.gov/projects/SNP/snp_ref.cgi?rs=.) | 36998203 | RegF | CTCF_binding_site | C | - | - | T | T | T | T | T |
| Trim15 | | [.](http://www.ncbi.nlm.nih.gov/projects/SNP/snp_ref.cgi?rs=.) | 36998241 | RegF | CTCF_binding_site | A | - | - | C | C | C | C | C |
| Trim15 | | [.](http://www.ncbi.nlm.nih.gov/projects/SNP/snp_ref.cgi?rs=.) | 36998248 | RegF | CTCF_binding_site | T | - | - | C | C | C | C | C |
| Trim15 | | [.](http://www.ncbi.nlm.nih.gov/projects/SNP/snp_ref.cgi?rs=.) | 36998254 | RegF | CTCF_binding_site | T | - | - | C | C | C | C | C |
| Trim15 | | [.](http://www.ncbi.nlm.nih.gov/projects/SNP/snp_ref.cgi?rs=.) | 36998266 | RegF | CTCF_binding_site | A | - | - | G | G | G | G | G |
| Trim15 | | [.](http://www.ncbi.nlm.nih.gov/projects/SNP/snp_ref.cgi?rs=.) | 36998287 | RegF | CTCF_binding_site | A | - | - | C | C | C | C | C |
| Trim15 | | [.](http://www.ncbi.nlm.nih.gov/projects/SNP/snp_ref.cgi?rs=.) | 36998326 | RegF | CTCF_binding_site | A | - | - | G | G | G | G | G |
| Trim15 | | [.](http://www.ncbi.nlm.nih.gov/projects/SNP/snp_ref.cgi?rs=.) | 36998329 | RegF | CTCF_binding_site | C | - | - | T | T | T | T | T |
| Trim15 | | [.](http://www.ncbi.nlm.nih.gov/projects/SNP/snp_ref.cgi?rs=.) | 36998401 | RE | H3K36_trimethylation_site | G | - | - | A | A | A | A | A |
| Trim15 | | [.](http://www.ncbi.nlm.nih.gov/projects/SNP/snp_ref.cgi?rs=.) | 36998499 | RE | H3K36_trimethylation_site | A | - | - | G | G | G | G | G |
| Trim15 | | [.](http://www.ncbi.nlm.nih.gov/projects/SNP/snp_ref.cgi?rs=.) | 36998519 | RE | H3K36_trimethylation_site | C | - | - | T | T | T | T | T |
| Trim15 | | [.](http://www.ncbi.nlm.nih.gov/projects/SNP/snp_ref.cgi?rs=.) | 37002866 | RE | H3K27_trimethylation_site | T | - | - | C | C | C | C | C |
| Trim15 | | [.](http://www.ncbi.nlm.nih.gov/projects/SNP/snp_ref.cgi?rs=.) | 37003025 | RE | H3K27_trimethylation_site | A | - | - | G | G | G | G | G |
| Trim15 | | [.](http://www.ncbi.nlm.nih.gov/projects/SNP/snp_ref.cgi?rs=.) | 37004408 | RE | H3K27_trimethylation_site | A | - | - | C | C | C | C | C |
| Trim15 | | [rs6165728*](http://www.ncbi.nlm.nih.gov/projects/SNP/snp_ref.cgi?rs=rs6165728) | 37004408 | Motif | CREB1 | C | A | - | C | C | - | C | C |
| Trim15 | | [.](http://www.ncbi.nlm.nih.gov/projects/SNP/snp_ref.cgi?rs=.) | 37004438 | RE | H3K27_trimethylation_site | G | - | - | A | A | A | A | A |
| Trim10 | | [.](http://www.ncbi.nlm.nih.gov/projects/SNP/snp_ref.cgi?rs=.) | 37010757 | RE | H3K36_trimethylation_site | G | - | - | T | T | T | T | T |

Candidate SNPs were obtained by searching the ECCO database[41]. This table shows SNPs that affect regulatory sites and whose alleles are shared by B6, 129 and CAST strains, but are different in all the other CC founder strains. Experimental evidence is denoted as follows: RE, regulatory evidence; RegF = regulatory feature[42]. SNPs indicated with an asterisk were identified by the FANTOM5 project.

| **Supplementary Table 5. Strain sample numbers** | | | | | |
| --- | --- | --- | --- | --- | --- |
| **Strain** | **8-12 months** | | **>12 months** | | **Total** |
|  | **M** | **F** | **M** | **F** |  |
| BEW | 0 | 0 | 4 | 2 | 6 |
| BEM | 3 | 3 | 0 | 0 | 6 |
| BOON | 3 | 1 | 0 | 2 | 6 |
| CAMERON | 0 | 2 | 0 | 1 | 3 |
| CIS | 0 | 0 | 2 | 6 | 8 |
| CIS2 | 0 | 0 | 2 | 2 | 4 |
| CIV2 | 0 | 0 | 1 | 1 | 2 |
| DAVIS | 0 | 0 | 3 | 2 | 5 |
| DET3 | 2 | 2 | 0 | 1 | 5 |
| DONNELL | 0 | 0 | 3 | 3 | 6 |
| FUF | 0 | 1 | 2 | 2 | 5 |
| GALASUPREME | 0 | 1 | 2 | 2 | 5 |
| GAV | 3 | 3 | 0 | 0 | 6 |
| GEK2 | 3 | 3 | 0 | 0 | 6 |
| GET | 0 | 0 | 1 | 3 | 4 |
| HAX2 | 0 | 3 | 0 | 0 | 3 |
| HAZ | 3 | 3 | 0 | 0 | 6 |
| HIP | 0 | 4 | 1 | 1 | 6 |
| HOE | 1 | 0 | 0 | 3 | 4 |
| JAFFA | 0 | 0 | 2 | 2 | 4 |
| JEUNE | 0 | 3 | 0 | 0 | 3 |
| JUD | 1 | 1 | 1 | 3 | 6 |
| JUNIOR | 0 | 0 | 2 | 2 | 4 |
| MAK | 0 | 0 | 3 | 0 | 3 |
| MEE | 0 | 0 | 1 | 1 | 2 |
| MERCURI | 0 | 0 | 2 | 0 | 2 |
| MOK | 0 | 0 | 1 | 1 | 2 |
| MOP | 2 | 5 | 1 | 1 | 9 |
| PEF | 2 | 3 | 0 | 0 | 5 |
| PEF2 | 0 | 0 | 3 | 4 | 7 |
| PER2 | 1 | 2 | 1 | 1 | 5 |
| PIPING | 3 | 3 | 0 | 0 | 6 |
| POH | 1 | 2 | 2 | 1 | 6 |
| POT | 0 | 0 | 1 | 0 | 1 |
| RAE2 | 0 | 0 | 2 | 6 | 8 |
| REV | 0 | 0 | 0 | 4 | 4 |
| ROGAN | 0 | 0 | 3 | 4 | 7 |
| SAT | 1 | 2 | 1 | 1 | 5 |
| SOLDIER | 0 | 0 | 1 | 3 | 4 |
| STUCKY | 2 | 1 | 1 | 0 | 4 |
| TAS | 0 | 1 | 0 | 0 | 1 |
| TOFU | 1 | 2 | 1 | 1 | 5 |
| VIT | 3 | 2 | 0 | 0 | 5 |
| WAB2 | 0 | 0 | 2 | 3 | 5 |
| WAD | 0 | 2 | 0 | 1 | 3 |
| WOB2 | 1 | 2 | 1 | 0 | 4 |
| WOT2 | 0 | 0 | 1 | 8 | 9 |
| YID | 3 | 3 | 0 | 0 | 6 |
| ZIF2 | 2 | 0 | 0 | 3 | 5 |
| ZOE | 0 | 0 | 1 | 0 | 1 |
| **Total** |  | **101** |  | **136** | **237** |
| Total samples deemed unfit for use |  |  |  |  | 38 |
| **Total samples** |  |  |  |  | **275** |
