# Supplementary material for: Age-dependent genetic regulation of osteoarthritis: independent effects of immune system genes
Source: Arthritis Res Ther. 2023 Dec 1;25:232. doi: 10.1186/s13075-023-03216-2 (PMC10691153; doi:10.1186/s13075-023-03216-2)
Supplement: Supplementary file 2 — Additional file 2. Supplementary Methods. [file 13075_2023_3216_MOESM2_ESM.docx]

**SUPPLEMENTARY METHODS**

The Collaborative Cross Gene Mine was generated by Grant Morahan at Geniad. Eight founder strains, A/J, C57BL/6J, 129S1/SvImJ, NOD/ShiLtJ, NZO/H1LtJ, CAST/EiJ, PWK/PhJ and WSB/EiJ, (these will be referred to throughout the results as A/J, C57BL/6J, 129, NOD, NZO, CAST, PWK and WSB). (G0) are crossed to generate the first generation (G1), which are then bred to generate the parent strain (G2). The offspring of the parent strain (G3) are considered to have a contribution of all eight founder strains (G0). Mice generated from G3 are then inbred for a minimum of 20 generations at which point they are considered fully inbred. Strains generated at G23 and over are each given identifiers used to classify mice under recombination headings. Mice under each heading will then be culled and have their hind limbs removed for analysis. The use of all animals in this study was conducted in accordance with the appropriate ethics approvals and the Australian Code for the Care and Use of Animals for Scientific Purposes.

Geniad have over 100 CC strains breeding and available beyond G15 [1]. Genotyping of the founder strains and CC strains have been conducted via the MegaMUGA array by typing 77,808 SNPs throughout the founder genomes, to provide genome-wide coverage. The initial power calculations of the CC demonstrated that the use of 500 strains provided a detection power of 67% to detect a QTL with an additive effect of 5%. Detection power reached approximately 100% when the effect of the QTL exceeded 10% [2]. The ability to generate and test 500 strains is however unrealistic, therefore the majority of studies thus far have utilised less than 100 strains for screening and mapping loci [3, 4]. Ram et al. has demonstrated identification of genes using small strain numbers as a proof of principle [5]. This study utilised a total number of 291 Geniad mice and 50 strains for phenotype screening. After the exclusion of poor tissue condition and strains without replicates the total numbers used for QTL mapping are 222 Geniad mice and 43 strains.

The genotypes for the eight founders were obtained via the University of North Carolina CC website (<http://csbio.unc.edu/CCstatus/index.py?run=GeneseekMM>). The genotypes of the founders were separated into two sets per strain, homozygous genotypes of allele 1 and homozygous genotypes of allele 2 for their genomes to be considered haploid or fully inbred. Researchers at Geniad, led by Prof Grant Morahan inputted this data into HAPPY software using a method called “hdesign” [5, 6] which is designed to estimate the founder haplotype having the maximum likelihood probability for genotype sets of allele 1 and 2 independently. After verification of the haplotype data, users are able to interface with the dataset via online software called Geneminer (<https://www.sysgen.org/Geniad2/>). This software has been developed for the purpose of correlating phenotypes observed in the CC mice with their genotypes for the purpose of gene mapping.

The process of gene mapping is carried out on the Geneminer software. In this study, strains are ranked via numerical values in accordance with the observed phenotype. In this case, each strain is given the median values of OARSI scores observed in their respective phenotype classifications or percentage values given for incidence of phenotypes per CC strain. This data is inputted into Geneminer (version: 19/05/2016) to compare haplotypes of the ranked strains and identify loci that are represented by founder contributions that are unique to the highly ranked CC strains. The QTL analysis is carried out in raw data format (or “as is”) and normalized data format. Each run generates a QTL plot for each respective parameter or phenotype. The purpose of normalisation when running the QTL analysis is for the optimisation of asymmetrical data distribution. Strains scores are ranked from highest to lowest with the majority of the cohort falling within a central bell curve style distribution. In cases where the data distribution is weighted towards higher or lower scores, a normalization function can be selected to clearly define the differences between high scoring strains and the remainder of the cohort.

The data output from Geneminer is represented in the form of a plot showing peaks across the genome measured in LOD values. These plots demonstrate the correlation between the founder SNP markers and the phenotype observed within the strains. This correlation produces peak(s) within chromosome(s), which are interpreted as significant based on the LOD score and passing pre-set thresholds within the software. The thresholds for significance are calculated from 1,000 additive model permutations and set by Geniad and Prof Grant Morahan. This method was constructed based on the approach recommended by [7] and has been applied by groups such as [4]. These thresholds are represented by two lines: a red line indicating the 95^th^ percentile of confidence which is equivalent to p <0.05, and a yellow line representing the 37^th^ percentile of confidence which is equivalent to p <0.63. Peaks that cross the 95^th^ percentile are considered as genome wide significant, while peaks crossing only the 37^th^ percentile are considered to be suggestive only.

Gene investigation is carried out by selecting QTL peaks of interest, and observing the Geneminer output from the genomic interval selection. The output will present SNPs present from founders contributing to the selected interval. In addition to the Geneminer SNPs, the ENCODE database will provide a more detailed selection of SNPs including the missense/regulatory effects present at the selected locus by inputting the interval and founders that are contributing to the site.

The Geneminer software calculates the contribution of each founder to each observed trait. This is determined by coefficients (log of odds ratio) of the fit from the logistic/multinominal regression model and using the plotting tools in the DOQTL R package [8]. The founder effects calculated within the system are represented by p-values to represent the dominant founder contributions to each QTL. The SNPs derived from the prominent founder/s at the QTL in question will be considered as the significant contributor to the phenotype. This founder coefficient is of importance when considering genes implicated at a QTL of interest that are contributed by many evenly distributed founders. When many founders are contributing to a single locus, there is a decreased likelihood of the genes implicated at that locus to be considered strong candidates.

**References**

1. Morahan, G., L. Balmer, and D. Monley, *Establishment of "The Gene Mine": a resource for rapid identification of complex trait genes.* Mamm Genome, 2008. **19**(6): p. 390-3.

2. Valdar, W., J. Flint, and R. Mott, *Simulating the collaborative cross: power of quantitative trait loci detection and mapping resolution in large sets of recombinant inbred strains of mice.* Genetics, 2006. **172**(3): p. 1783-97.

3. Levy, R., et al., *Collaborative cross mice in a genetic association study reveal new candidate genes for bone microarchitecture.* BMC Genomics, 2015. **16**: p. 1013.

4. Boutilier, J.K., et al., *Variable cardiac α-actin (Actc1) expression in early adult skeletal muscle correlates with promoter methylation.* Biochimica et Biophysica Acta (BBA) - Gene Regulatory Mechanisms, 2017. **1860**(10): p. 1025-1036.

5. Ram, R., et al., *Rapid identification of major-effect genes using the collaborative cross.* Genetics, 2014. **198**(1): p. 75-86.

6. Mott, R., et al., *A method for fine mapping quantitative trait loci in outbred animal stocks.* Proceedings of the National Academy of Sciences, 2000. **97**(23): p. 12649-12654.

7. Durrant, C., et al., *Collaborative Cross mice and their power to map host susceptibility to Aspergillus fumigatus infection.* Genome Research, 2011. **21**(8): p. 1239-1248.

8. Gatti, D.M., et al., *Quantitative trait locus mapping methods for diversity outbred mice.* G3: Genes, Genomes, Genetics, 2014. **4**(9): p. 1623-1633.
